# Intricately Regulated: A Cellular Toolbox for Fine-Tuning XBP1 Expression and Activity

**DOI:** 10.3390/cells1040738

**Published:** 2012-10-15

**Authors:** Andrew E. Byrd, Joseph W. Brewer

**Affiliations:** Department of Microbiology and Immunology, College of Medicine, University of South Alabama, Mobile, AL 36688, USA; Email: eab303@jaguar1.usouthal.edu

**Keywords:** endoplasmic reticulum, endoplasmic reticulum stress, unfolded protein response, XBP1

## Abstract

Stress in the endoplasmic reticulum (ER) triggers the unfolded protein response (UPR), a signaling mechanism that allows cellular adaptation to ER stress by engaging pro-adaptive transcription factors and alleviating protein folding demand. One such transcription factor, X-box binding protein (XBP1), originates from the inositol-requiring transmembrane kinase/endoribonuclease 1 (IRE1) UPR stress sensor. XBP1 up-regulates a pool of genes involved in ER protein translocation, protein folding, vesicular trafficking and ER- associated protein degradation. Recent data suggest that the regulation of XBP1 expression and transcriptional activity may be a tissue- and stress-dependent phenomenon. Moreover, the intricacies involved in “fine-tuning” XBP1 activity in various settings are now coming to light. Here, we provide an overview of recent developments in understanding the regulatory mechanisms underlying XBP1 expression and activity and discuss the significance of these new insights.

## 1. Introduction

Cells are highly sensitive to conditions that disrupt the environment of the endoplasmic reticulum (ER) or that increase demand on its machinery for synthesis, maturation and transport of secretory cargo. Various physiological and pathophysiological conditions interfere with protein folding processes in the ER, leading to accumulation of misfolded or unfolded proteins, a cellular condition referred to as “ER stress”. Adapting to the nature, intensity, and duration of ER stress, cells launch the unfolded protein response (UPR), a tri-partite intracellular signaling mechanism that triggers translational control and an extensive transcriptional response to balance client protein load with the folding capacity of the ER [1]. The three distinct mammalian UPR signaling pathways are initiated by the ER transmembrane sensors protein kinase RNA-activated (PKR)-like ER kinase (PERK), activating transcription factor 6 (ATF6), and inositol-requiring enzyme 1 (IRE1). Activated PERK phosphorylates the α subunit of eukaryotic initiation factor 2 (eIF2α), effectively down-regulating protein synthesis [2,3,4,5]. Proteolytic processing of ATF6 yields an active transcription factor (ATF6α(N)) that up-regulates expression of ER resident quality control proteins, including chaperones and ER-associated degradation (ERAD) components [6,7,8,9,10]. Upon activation of IRE1, its endoribonucleolytic activity catalyzes an unconventional cytosolic splicing of X-box binding protein 1 (*XBP1*) mRNA, resulting in a translational frameshift that generates XBP1S, a pro-adaptive, basic-region leucine zipper (bZIP) transcription factor [11,12,13]. If these adaptive mechanisms do not sufficiently recover ER homeostasis, the UPR can commit the damaged cell to death by apoptosis [14].

XBP1 is subject to transcriptional, post-transcriptional and post-translational control mechanisms [15,16,17,18,19,20,21,22,23,24,25,26], indicating that the activity of this crucial UPR transcription factor is carefully balanced. Indeed, XBP1 has tissue-distinct roles during the UPR in different physiological processes and diseases. XBP1, a member of the CREB/ATF (cyclic AMP response element binding protein/activating transcription factor), family of transcription factors, was originally identified as a protein that binds to the HLA (human leukocyte antigen) DRα promoter [27]. Since then, studies have unveiled multiple roles for XBP1 in diverse processes including secretory function, the inflammatory response, lipid metabolism and glucose homeostasis [28]. XBP1 deficiency impairs normal development and function of antibody-producing plasma cells [29] as well as pancreatic, liver and salivary gland cells [30,31,32,33]. XBP1 also plays a role in the development and survival of dendritic cells [34] and in the response of macrophages to certain Toll-like receptor agonists [35]. Defects in the XBP1 gene and/or protein have also been linked to critically important problems in human health such as ulcerative colitis/Crohn’s disease [36], insulin resistance and type 2 diabetes [37]. 

On the whole, these observations highlight the critical role of XBP1 as a pro-adaptive transcription factor in various physiological processes. Though genetically modified mice have allowed key discoveries of XBP1 function, a better understanding is needed concerning the mechanisms employed by cells to specifically moderate the activity of XBP1. 

## 2. XBP1 Biosynthesis and Function

**Figure 1:**
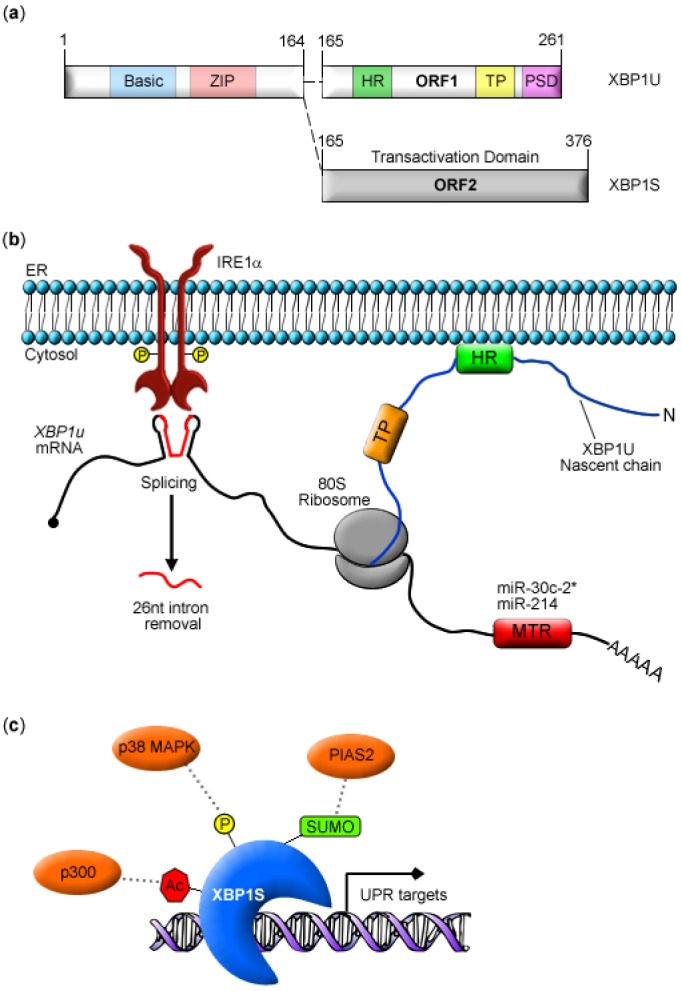
Regulatory elements of XBP1 expression and activity.**(a)** Figure of XBP1 protein variants (XBP1U and XBP1S). Numbers indicate amino acid positions. Basic and leucine zipper (ZIP) domains are indicated as well as ORF1 and ORF2 in the carboxyl-terminal domain of XBP1U and XBP1S, respectively. Hydrophobic region (HR), translational pausing (TP) and “proteasome-susceptible” degradation (PSD) domains of XBP1U are also highlighted. **(b)** Model for *XBP1* mRNA localization and processing at the ER membrane. The ER-localizing HR domain, TP domain, and microRNA targeting regions (MTR) are highlighted. The 26 nt intron spliced from *XBP1u* mRNA by IRE1α is depicted in red. **(c)** Post-translational modifications of XBP1S. Acetylation (Ac) by p300 increases stability and enhances transcriptional activity of XBP1S. SUMOylation (SUMO) by PIAS2 decreases activity of XBP1S. p38 MAPK-mediated phosphorylation (P) enhances XBP1S nuclear localization.

IRE1 is a bifunctional, single-pass ER transmembrane Ser/Thr kinase and endoribonuclease. This UPR transducer was originally identified in yeast [38,39,40] and is very similar across eukaryotic species. Mammals express two isoforms of IRE1, α and β. IRE1α is ubiquitously expressed [41], whereas IRE1β is limited to the gut epithelium [42]. Here, our discussion will focus on IRE1α since it is expressed in all cell types. In cells experiencing ER stress, active IRE1α catalyzes the cytosolic removal of a 26 nucleotide (nt) intron from XBP1 pre-mRNA (*XBP1u*) (Figure 1b). This unconventional splicing event results in a translational frameshift that joins the two open reading frames (ORFs) encoding a DNA binding domain (DBD) and a transcriptional activation domain (AD) [11,12,13] (Figure 1a). Translation of spliced *XBP1* mRNA (*XBP1s*) generates XBP1S, a bZIP transcription factor that binds to and activates two *cis*-acting promoter motifs, the ER stress response element (ERSE) and unfolded protein response element (UPRE) [13]. In addition to activating its own transcription, XBP1S induces genes encoding: factors involved in translocation of nascent polypeptides into the ER (SRP54, SEC61A, SEC61G and TRAM1); ER chaperones (ERdj4, ERdj5, HEDJ, GRP58 and PDI-P5), ERAD components (EDEM, OS9, HERP, and p58^IPK^); factors involved in vesicular transport (SEC23B and SEC24C)) [43,44]. More recently, XBP1S was shown to up-regulate expression of a microribonucleic acid (miRNA), miR-346, that targets the human antigen peptide transporter 1 (TAP1) mRNA [45]. 

Complete deletion of XBP1 in mice results in embryonic lethality at ~13 weeks gestation [33]. Rescue of embryonic lethality by targeting an XBP1 transgene selectively to hepatocytes led to early post-natal lethality via activation of ER stress-mediated proapoptotic pathways [30]. Specifically, the phenotype consisted of weak expression of ER chaperone genes and poorly developed ER in pancreatic and salivary gland acinar cells, and this correlated with impaired production of pancreatic digestive enzymes [30]. Similarly, XBP1S is vital for ER expansion and induction of high-rate immunoglobulin synthesis during plasma cell differentiation [44,46]. 

## 3. Post-transcriptional Modulation of XBP1 Expression

Recent reports indicate that post-transcriptional mechanisms influence the fate of *XBP1* mRNA. Regulatory mechanisms implicated include unique localization of *XBP1* mRNA at the ER membrane and translational pausing that facilitates IRE1α-dependent splicing. In addition, *XBP1* mRNA is targeted by miRNA. 

IRE1α-mediated splicing of *XBP1* mRNA occurs in the cytosol [47,48], in contrast to conventional mRNA splicing that takes place in the nucleus. Only recently have discoveries shed light on underlying mechanisms that orchestrate the localization of *XBP1* mRNA within proximity of IRE1α at the ER membrane (Figure 1b). A novel observation of cellular localization of total *XBP1* mRNA was reported in a study examining mRNA partitioning and translation in the ER and cytosolic compartments during the UPR [49]. Surprisingly, total *XBP1* mRNA was found to be predominantly membrane associated, although its protein products, XBP1U and XBP1S, are soluble [49]. A subsequent study confirmed *XBP1u* mRNA association with the ER membrane, but reported *XBP1s* mRNA re-distribution to cytosolic compartments for translation [24]. Yanagitani and colleagues [24] further implicated a conserved hydrophobic region (HR2) near the carboxyl-terminus of XBP1U as an ER membrane association domain (Figure 1a, b). This group speculated that the HR2 of nascent XBP1U polypeptide chains might cotranslationally recruit *XBP1u* mRNA to the ER membrane as part of a mRNA-ribosome-nascent chain complex (R-RNC) [24] (Figure 1b). In addition, they recently reported that translation of the *XBP1u* mRNA transiently pauses to stabilize the R-RNC complex [25]. This entire process is dependent on XBP1U sequences that are highly similar across multiple species, specifically the HR2 and an additional region near the carboxyl-terminus [25] (Figure 1a). 

While the Stephens [49] and Yanagitani [24,25] studies agree that *XBP1u* mRNA localizes at the ER membrane, ambiguity remains as to whether *XBP1* mRNA shifts from the ER membrane to the cytosol after IRE1α-mediated splicing has occurred. Notably, the two studies were conducted in different cell lines under different strengths of ER stress inducers. Importantly, the HR2 is located within the 3’ segment of the *XBP1u* coding region where the translational frame is altered by IRE1α−mediated splicing, resulting in XBP1S which lacks the HR2 [24]. Finally, studies of XBP1-deficient mice have revealed hyperactivation of IRE1α associated with splicing of a truncated *XBP1* mRNA in liver and intestinal tissue [32,36], indicating that expression of XBP1U is not required for splicing. Perhaps, the sub-cellular distribution of total *XBP1* mRNA is determined in a tissue- and/or stress-specific fashion. Further studies are required to delineate a full understanding of these mechanisms and their relevance *in vivo*.

More recently, we and others have reported miRNA-mediated regulation of XBP1 expression via the 3’ untranslated region (UTR) of *XBP1* mRNA [15,50] (Figure 1b). miRNA are a class of endogenous, non-coding, single-stranded RNAs ~22 nts long that typically function as post-transcriptional repressors of gene expression [51]. Although the specific biological functions of miRNA in ER stress and the UPR remain largely unknown, a few ER stress-inducible miRNAs have been identified [15,45,52].

Our group identified a miRNA, miR-30c-2* (since designated miR-30c-2-3p), that targets a single site in the 3′-UTR of XBP1 mRNA (Figure 1b). Over-expressing miR-30c-2* reduced the levels of XBP1 and its target genes in stressed cells, whereas inhibiting miR-30c-2* activity had the opposite effect, boosting XBP1 levels and promoting cell survival [15]. Induction of ER stress by subjecting human and mouse cell lines to treatment with tunicamycin (Tm), an inhibitor of *N*-linked glycosylation, triggers the UPR and activates IRE1α, thereby generating XBP1S. Interestingly, miR-30c-2* is up-regulated during the Tm-induced UPR, concomitant with XBP1, suggesting that this miRNA might affect XBP1S expression levels as the UPR proceeds [15]. Our study further showed that up-regulation of miR-30c-2* is dependent on another key signaling component of the UPR, the protein kinase PERK. The mechanism involves the transcription factor nuclear factor-κB (NF-κB) and its interaction with a NF-κB enhancer motif upstream of the miR-30c-2* genes [15]. In the UPR, NF-κB is activated downstream of PERK [53,54]. Importantly, a separate study revealed that phosphorylation of eIF2α is required for optimal induction of XBP1S by a mechanism involving both *XBP1* mRNA stabilization and translation inhibition [18]. Therefore, emerging evidence indicates that regulatory cross-talk between the IRE1/XBP1 and PERK pathways influences the strength of XBP1S induction.

Another miRNA, miR-214, was recently implicated as a negative regulator of XBP1 expression in hepatocellular carcinomas (HCC) [50] (Figure 1b). In HCC, miR-214 expression was down-regulated in response to pharmacologic ER stress-inducing agents, hypoxia and lipopolysaccharide stimulation, suggesting that miR-214 represses XBP1 expression until the UPR is activated. Down-regulation of miR-214 correlated with NF-κB activation, whereby NF-κB exerted a negative regulatory affect on expression of the miR-199a/214 gene cluster [50]. However, a direct binding site through which NF-κB mediates this effect was not demonstrated, raising the possibility of an indirect regulatory mechanism. 

Together, these findings break the ice for exploration into potential roles for miRNA-mediated regulation of XBP1. However, the *in vivo* relevance of these mechanisms remains unexplored. It will be interesting to investigate the degree to which miRNA regulate XBP1 translation and affect gene expression, cell function and cell fate in various physiological and pathophysiological conditions involving XBP1, such as plasma cell differentiation [46], macrophage activation [35] and tumor cell survival [55].

## 4. Post-translational Modifications of XBP1S

A series of recent discoveries have implicated XBP1S as a target for a variety of post-translational modifications, including phosphorylation, SUMOylation, acetylation and deacetylation (Figure 1c). These modifications affect XBP1S turnover, sub-cellular localization and transcriptional activity. 

### 4.1. Phosphorylation

A recent study revealed a novel phosphorylation of XBP1S and linked this modification to the maintenance of glucose homeostasis in mice [17]. Convincing evidence showed that XBP1S is phosphorylated on Thr^48^ and Ser^61^ by the ER stress responsive p38 MAPK (mitogen-activated protein kinase) (Figure 1c) and that this modification enhanced its nuclear translocation by 5-fold [17]. The study further addressed whether p38 MAPK modulates XBP1S activity *in vivo*. In the *ob/ob* mouse model of obesity, reduced XBP1S activity in the liver has been associated with increased liver tissue-specific ER stress and defective glucose homeostasis [20]. These observations correlate well with defective XBP1S nuclear translocation in the livers of *ob/ob* mice [20]. Indeed, inhibition of p38 MAPK blocked entry of XBP1S into the nucleus, whereas restoration of p38 MAPK activity in the livers of *ob/ob* mice enhanced XBP1S nuclear translocation [17]. These data highlight the significance of XBP1S phosphorylation on Thr^48^ and Ser^61^ for its nucleocytoplasmic shuttling and demonstrate the impact of this event on cell function *in vivo*. Perhaps, this novel mechanism of XBP1S post-translational modification via phosphorylation can be exploited for therapeutic treatment of various pathological states including obesity and type 2 diabetes, inflammatory conditions, and multiple myeloma.

### 4.2. SUMOylation

The post-translational modification of proteins by small ubiquitin-like modifier (SUMO) has been shown to regulate a variety of cellular processes such as signal transduction, protein degradation, nuclear-cytosolic transport and gene transcription [56]. A seminal study showed that two lysine residues, Lys^276^ and Lys^297^, near the carboxyl-terminus of XBP1S are subject to covalent SUMOylation [16]. In addition, XBP1S SUMOylation appears to be highly specific for the SUMO E3 ligase PIAS2 (protein inhibitor of activated STAT2) (Figure 1c). These two SUMOylation events exerted an additive negative effect on XBP1S, reducing its transcriptional activity. Conversely, ablation of SUMOylation significantly enhanced XBP1S transcriptional activity. The study further demonstrated that SUMOylation does not alter nuclear translocation of XBP1S [16]. Thus, the mechanism by which SUMOylation dampens XBP1S activity and the significance of this mechanism in physiologic processes involving XBP1S await further investigation. 

### 4.3. Acetylation and Deacetylation

XBP1S can also be targeted for acetylation and deacetylation mediated by p300 and SIRT1 (sirtuin 1), respectively [22]. As p300 increases acetylation of XBP1S, the stability and transcriptional activity of XBP1S are enhanced (Figure 1c). In contrast, SIRT1 deacetylates XBP1S and inhibits its transcriptional activity (Table 1). Consistent with these data, SIRT1^−/−^ MEFs (mouse embryo fibroblasts) subjected to pharmacologic ER stress exhibited greater resistance to ER stress-induced cell death when compared to wild-type MEFs [22]. Therefore, the extent that acetylation and deacetylation influence XBP1S in physiologic settings warrants further study.

## 5. Protein-protein Interactions of XBP1U and XBP1S

**Figure 2:**
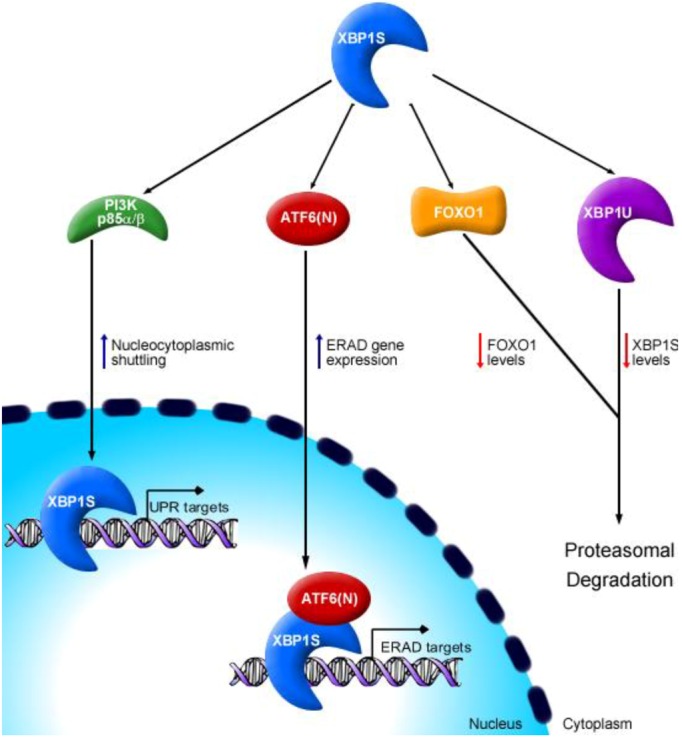
Binding partners of XBP1 proteins. The transcriptional activity of XBP1S is modulated through protein-protein interactions. The p85α/β subunits of phosphatidylinositol 3-kinase (PI3K) bind to XBP1S and enhance its nuclear translocation. The active form of activating transcription factor 6 that traffics into the nucleus (ATF6(N)) interacts with XBP1S to exert a combinatorial effect on expression of ER-associated degradation (ERAD) components. Proteasome-mediated degradation of XBP1S is enhanced by direct interactions with both XBP1U and the transcription factor Forkhead box O1 (FOXO1).

A number of binding partners have recently been identified for the XBP1 proteins, including transcription factors and other types of regulatory proteins. Such protein-protein interactions have been implicated in regulating the turnover, nucleocytoplasmic shuttling and transcriptional activity of XBP1S (Figure 2). 

### 5.1. XBP1U Negatively Regulates XBP1S

The role(s) of XBP1U have not been easily identified. However, Yoshida and colleagues [26] made a key discovery in showing that heterodimerization of XBP1U and XBP1S negatively regulates XBP1S. XBP1U contains a DBD, lacks an AD and is extremely labile [11,13,19,21,57]. In contrast, while XBP1S contains the same DBD, it also harbors an AD and is more stable [11]. However, both XBP1U and XBP1S contain a nuclear localization sequence (NLS) required for nuclear translocation. In addition to the NLS, XBP1U contains two other subcellular localization determinants: a nuclear export sequence (NES) and a NES attenuator [26]. As XBP1U shuttles between the nucleus and the cytoplasm, this factor can bind XBP1S and promote its localization to the cytoplasm [26]. This mechanism appears to facilitate proteasomal degradation of the XBP1U-XBP1S complex (Figure 2). Supporting this idea is the presence of a proteasome-susceptible “degradation domain” located in amino acids 209-261 of XBP1U [26] and recent evidence that XBP1U interacts with 20S proteasomes via its carboxyl-terminus [19]. Though this model warrants further investigation, particularly in the context of physiologic processes, XBP1U appears to function as a repressor of XBP1S activity as the UPR proceeds. This mechanism might play an especially significant role under conditions in which UPR-mediated splicing of *XBP1* mRNA is either weakly activated or is waning due to resolution of ER stress. 

### 5.2. p85 Subunit of PI3K Enhances XBP1S Nucleocytoplasmic Shuttling

More recently, two independent studies were simultaneously reported that identified an interaction between the p85α subunit of phosphatidylinositol 3-kinase (PI3K) and XBP1S in the context of metabolic control in diabetes models [20,23]. While both studies utilized *in vitro* model systems to validate the p85α-XBP1S interaction, Park and colleagues [20] went further to show that the p85β subunit could also form a protein complex with XBP1S. The p85-XBP1S interaction most notably increased XBP1S nucleocytoplasmic shuttling, resulting in increased nuclear accumulation of XBP1S [20,23] (Figure 2). Whether the reported nuclear accumulation is due strictly to enhanced nuclear transport, or whether p85 alters XBP1S stability needs further investigation. p85α and p85β also heterodimerize with each other to form an insulin-sensitive complex that dissociates when cells are exposed to insulin [20], an inducer of PI3K activity. Insulin stimulation also increases XBP1S nuclear translocation, suggesting a model whereby insulin facilitates the dissociation of the p85α-p85β complex, freeing the subunits to interact with XBP1S and enhance its nucleocytoplasmic shuttling [20] (Figure 2). In addition, p85α-deficient brown preadipocyte cells exhibited a defective Tm-induced UPR, including a 40% reduction in nuclear XBP1S relative to wild-type cells [23]. Winnay and colleagues [23] showed that liver-specific deletion of p85α in mice hindered the UPR, XBP1S nucleocytoplasmic shuttling and the ability of the liver to adequately resolve Tm-induced ER stress. Similar observations were made when mice lacking both p85α and p85β in the liver were subjected to refeeding-induced metabolic overload [20]. In insulin resistant *ob/ob* mice, refeeding-induced metabolic overload was accompanied by defective XBP1S nuclear translocation and diminished induction of ER chaperone genes in liver cells. Notably, this correlated with a reduced level of p85-XBP1S complexes [20]. In contrast, in livers of wild-type mice, XBP1S interacted with p85 subunits and a normal UPR was detectable [20]. These data suggest that p85-XBP1 complexes form *in vivo* and that this interaction is crucial for metabolic homeostasis in the liver. Specifically, it seems that an intact insulin-sensitive p85 dissociative response is required for the partnering of p85α and p85β with XBP1S, an interaction that optimizes nuclear translocation of XBP1S and ensures an appropriate UPR in the liver during conditions of metabolic overload. 

### 5.3. XBP1S Directs FOXO1 to Proteasome-Mediated Degradation

XBP1S was also reported to physically interact with the transcription factor Forkhead box O1 (FOXO1) [58] (Figure 2). The FoxO subfamily of transcription factors regulates assorted cellular functions, such as differentiation, metabolism, proliferation and survival [59,60]. FOXO1 is a wide-range regulator of energy homeostasis, including hepatic glucose output, adipocyte and muscle differentiation, and feeding behavior in the brain [59,60]. Importantly, FOXO1 promotes development of hyperglycemia in insulin-resistant conditions. XBP1S appears to bind directly to FOXO1 and facilitate its degradation by the 26S proteasome [58]. In addition, enforced expression of a DNA-binding-defective mutant of XBP1S in the liver tissue of *ob/ob* mice increased glucose tolerance and reduced blood glucose levels [58]. Prior to this report, the relevance of XBP1 in metabolic homeostasis was generally assumed to involve its classical function of improving ER folding capacity, thereby enhancing insulin sensitivity. Remarkably, this study suggests that XBP1S may act to reduce serum glucose concentration and increase glucose tolerance independently of its role in transactivating ER stress responsive genes [58]. These findings open new avenues of study regarding the XBP1S-FOXO1 complex in metabolic disorders like type 2 diabetes and raise the intriguing possibility that XBP1S might also affect other cellular processes via interaction with as yet undefined regulatory factors. 

**Table 1:** Molecular mechanisms modulating XBP1 expression and activity. Overview of reported regulatory mechanisms for the XBP1 transcription factor.

Cellular Process	Effector	Mechanism	Effect on XBP1	Model	Reference
Post-trancription	IRE1	cytosolic mRNA splicing	cytosolic mRNA splicing/religation/ORF frameshift	pharmacologic-induced ER stress	[11,12,13]
	HR2 of nascent XBP1U	*XBP1u* mRNA recruitment	localize *XBP1* mRNA at ER membrane within close proximity of IRE1	*in vitro* expression of XBP1	[24]
	*XBP1u* COOH-terminus	translational pausing	stabilize R-RNC complex at ER membrane for efficient targeting and splicing of *XBP1u* mRNA	pharmacologic-induced ER stress	[25]
	miR-30c-2*	inhibits XBP1	fine-tune XBP1 expression as the UPR proceeds	pharmacologic-induced ER stress	[15]
	miR-214	expression inhibits XBP1	repress XBP1 expression prior to UPR activation	LPS- and pharmacologic- induced ER stress	[50]
Post-translation	p38 MAPK	phosphorylation	increase XBP1S stability/enhance XBP1S nucleocytoplasmic shuttling	pharmacologic-induced ER stress; *ob/ob* mice	[17]
	PIAS2	SUMOylation	decrease transcriptional activity of XBP1S	*in vitro* expression of XBP1; pharmacologic-induced ER stress	[16]
	p300	acetylation	increase XBP1S stability	pharmacologic-induced ER stress	[22]
	SIRT1	deacetylation	decrease XBP1S stability	pharmacologic-induced ER stress	[22]
Protein interaction	XBP1U	heterodimerization	target XBP1U-XBP1S complex for proteasome-mediated degradation	transient over-expression *in vitro*	[26]
	PI3K p85 subunits	heterodimerization	enhance XBP1S nucleocytoplasmic shuttling	pharmacologic-induced ER stress; metabolic induced overload in *ob/ob* mice	[20,23]
	FOXO1	heterodimerization	direct protein complex to 26S proteasome	*in vitro* expression; glucose homeostasis in *ob/ob* mice	[58]
	ATF6α( N)	heterodimerization	combinatorially enhance ERAD gene expression	pharmacologic-induced ER stress	[10]

### 5.4. ATF6α Interacts with XBP1S to Optimize ERAD

Another protein-protein interaction involves the combinatorial effect of XBP1S and the UPR transcription factor, ATF6α(N), in the expression of genes involved in ERAD. The transcriptional induction of ERAD components is a primary response to ER stress and aids in ER homeostasis by facilitating the elimination of terminally misfolded proteins. Both XBP1S and ATF6α(N) activate transcription of several ERAD genes via an as yet unidentified promoter element(s). However, *in vitro* studies revealed cross-talk between these two UPR branches, whereby ATF6α(N) heterodimerizes with XBP1S [10]. ATF6α cannot bind to the UPRE [13], a promoter element recognized by XBP1S and suspected to control expression of some ERAD genes. Thus, it was proposed that ATF6α-XBP1S heterodimers might regulate ERAD genes via the UPRE [10] (Figure 2). In support of this model, induction of some ERAD genes is dependent on both ATF6α and XBP1S [10]. A more recent study found that XBP1U can specifically bind to ATF6α(N) during the late-stage UPR and facilitate rapid degradation of both proteins [61]. This is consistent with a previous report that XBP1U can dimerize with ATF6α(N) [62], though the affect of the interaction was not established at the time. Both studies reported XBP1U interaction to be specific for ATF6α but not ATF4 [61,62], a transcription factor originating from the PERK branch of the UPR. Though these findings suggest a unique cross-talk between the ATF6α and IRE1/XBP1 branches of the UPR, further studies are needed to establish the relevance of these interactions *in vivo* during physiological and pathophysiological UPR-inducing events.

## 6. Concluding Remarks

Whether via transcriptional, post-transcriptional, co-translational, post-translational modifications and/or protein-protein interactions (Table 1), examples of mechanisms that can finely adjust XBP1S activity are increasingly evident. In the complexity of UPR signal transduction, the emergence of an IRE1/XBP1 signaling pathway that is subject to such diverse controls leads one to postulate that we have just begun to understand the breadth and importance of XBP1 regulatory activities. The combinatorial effects of these molecular mechanisms may vary widely depending on the tissue-type undergoing ER stress. For example, in the stressful conditions of the tumor microenvironment, different types of ER stressors and/or varying degrees of the same ER stress can be present, such as hypoxia and nutrient deprivation. 

An obvious, albeit challenging, direction for this exciting field is to establish the degree to which XBP1 regulatory mechanisms occur and function *in vivo*. Moving forward, considerable attention should be given to interpreting how these findings translate to our understanding of XBP1 related pathologies and the therapeutic potential of exploiting these mechanisms in certain disease states such as inflammatory disorders [63] and cancer [64]. Further defining the dynamic interplay of XBP1 and the molecular mechanisms that modulate its activity will provide a deeper understanding of the fundamental roles of the UPR, specifically IRE1/XBP1 signaling, in cell physiology.
